# Silanized Graphene Oxide/Glass Fiber-Modified Epoxy Composite with Excellent Anti-Corrosion and Mechanical Properties as Offshore Oil Platform Safety Signs

**DOI:** 10.3390/ma18091920

**Published:** 2025-04-24

**Authors:** Guanglei Lv, Peng Xiao, Yuhua Su, Xinmei Liu

**Affiliations:** 1 College of Chemistry and Chemical Engineering, China University of Petroleum (East China), Qingdao 266580, China; lvgl2@cnooc.com.cn (G.L.); xp894511832@163.com (P.X.); b21030034@s.up.edu.cn (Y.S.); 2 State Key Laboratory for Heavy Oil Processing, China University of Petroleum (East China), Qingdao 266580, China; 3 CNOOC (Tianjin) Pipeline Engineering Technology Co., Ltd., Tianjin 300450, China

**Keywords:** silanized oxidized graphene, silanized glass fiber, epoxy resin, mechanical and anti-corrosion properties

## Abstract

Epoxy resin (EP) is a candidate material for offshore oil platform safety signs due to its excellent corrosion resistance property. However, fabricating EP with good anti-corrosion as well as mechanical properties remains a significant challenge. Here, we report a new modification strategy to simultaneously improve the corrosion resistance and mechanical performance of EP by coupling it with KH550 silanized graphene oxide (KGO) and KH550 silanized glass fiber (KGF). KGO and KGF were grafted onto EP to obtain the modified EP material, i.e., KGO/KGF/EP composites and were characterized by FITR, XRD, SEM, and TGA to confirm the successful synthesis of the composites. It is shown that the tensile strength and adhesion strength of KGO/KGF/EP were 85.5 MPa and 16.0 MPa, which are 10.3% and 23.1% higher than KGO/GF/EP. Compared with KGF/EP, the corrosion potential increased by 9.9% and the corrosion rate decreased by 98.8%. Moreover, fluid–structure coupling simulation indicated the maximum stress of the material was within the criteria under extreme wind speeds, demonstrating its great potential for offshore oil platform safety sign applications.

## 1. Introduction

Safety signs are used to warn workers of dangerous conditions in the workplace or surrounding environment. These safety signs are installed with the support of base plate materials including stainless steel, aluminum, PVC, and acrylic plate. Unfortunately, they are expensive and not resistant to salt spray corrosion. Good anti-corrosion materials extend the service life of safety signs, ensure their visibility and identification, and guarantee structural stability and impact resistance. Epoxy resin (EP) is widely used as the basic anti-corrosion material due to its low toxicity, good mechanical, thermal, and barrier properties. However, EP is brittle and its anti-corrosion properties cannot meet the requirements of heavy anti-corrosion fields such as offshore oil safety platforms. EP needs to be modified before being used as the base plate material for safety signs.

At present, graphene oxide (GO) is mainly used to modify EP to improve its anti-corrosion properties [1,2,3]. Modifications of EP by GO include enhanced interfacial bonding and increased dispersion, improved toughness, strength, and thermal stability. In order to increase the adhesion and compatibility between GO and EP, GO is usually grafted with small molecules or polymers, and then added to EP. Previous reports used 3-aminopropyltriethoxy-silane and 3-ethoxy-propyltrimethoxysilane to modify graphene oxide before adding them to EP [2]. The modified GO can significantly improve the corrosion resistance of the composite material and increase the adhesion with the metal substrate by two times. Parhizkar et al. [4] used a silane coupling agent to covalently modify amino-functionalized GO film, which further improved the adhesion and anti-corrosion properties of composite material and metal substrate. He et al. [5] modified GO with tannic acid through a covalent bond with KH560 and added it to EP to obtain composite material, which significantly improved the corrosion resistance and permeability resistance of salt spray. Xiong et al. [6] used 3-aminopropyl triethoxysilane (APTES) to modify GO nanosheets and then added them to EP composites to study the effects of modified additives on the interface properties of composites and the compatibility of GO and EP composites. Experimental results showed that the composite exhibited the best corrosion resistance, crosslinking density as well as increasing the binding force with 0.5 wt.% of modified GO added to EP. Similarly, the use of polymer-modified GO to modify EP to improve its anti-corrosion properties has been extensively studied by scientific researchers. Pourhashem et al. [7] improved the dispersibility of GO in EP coating via modifying of GO with APTES. Chen et al. [8] prepared a sandwich-like structured material by using 8-hydroxyquinoline and polydopamine to modify GO with good corrosion resistance. Therefore, the incorporation of modified GO into EP is an effective method to enhance the corrosion resistance of the material.

Moreover, glass fiber (GF) has high specific strength, high modulus, low density, and high toughness [9], and it is widely used to improve the mechanical properties and corrosion resistance of matrix materials [10]. GF modifications to EP include interface modification enhancement, mechanical property optimization, and environmental resistance enhancement. 3-(2-Aminoethylamino) propyldimethoxymethylsilane modified graphene oxide (A-GO) and glass fiber (GF) were incorporated into epoxy (EP) matrix. The as-prepared EP/GF/A-GO composite showed that the highest impedance modulus compared with other coatings, indicating that the EP/GF/A-GO composite coating, with dual physical barrier network, could significantly enhance the corrosion protection properties. In addition, the EP/GF/A-GO composite coating has excellent anti-corrosion protection properties [11]. In addition, GF can improve the mechanical properties of EP as well. Mehmet et al. [12] infiltrated silicon carbide onto the surface of glass fiber by the process of infiltration and added it to EP for modification. In both cases (using fumed silica and multi-walled carbon nanotube (MWCNT)), the number of load cycles of the modified composites under tensile, alternating, and compressive loads increased by several orders of magnitude. These results may be attributed to the higher inter-fiber fracture strength of the composites. Rahman et al. [13] prepared epoxy composites reinforced with amino-functionalized MWCNT-modified E-glass woven fabric, which exhibited enhanced mechanical and thermomechanical properties. No improvement in mechanical properties was observed for these composites due to the weak interface between the glass fiber and the doped EP, which enhanced the detrimental effect of the accumulation bands of this GNPs [14]. However, it is difficult to improve the mechanical properties and anti-corrosion properties of EP at the same time by using a single modification method.

In the field of epoxy resin modification, the research on synergistic modification of silane coupling agent, GO, and GF is still in the exploratory stage, and most of the existing studies are focused on the optimization of a single material or a combination of two materials, with a lack of systematic research on the synergistic mechanism and performance balance of the three. Silane coupling agents are usually used to enhance the interfacial bonding between GF and epoxy resin, but the introduction of GO may interfere with the chemical bonding of the coupling agent, so we modified both. To explore the effect of synergistic modification of epoxy resins by silane coupling agents, GO, and GF, we chelated KH550-silanated graphene oxide (KGO) and KH550-silanated glass fiber (KGF) with EP. Unlike conventional physical blending, covalent grafting of silane groups (-NH_2_) on KGO and KGF establishes chemical bonding at the filler–substrate interface, which may result in a synergistic effect of corrosion resistance and mechanically enhanced stress transfer.

In this study, a multi-scale validation was carried out, Firstly, a series of characterization analyses (XRD, FITR) were conducted to confirm the synthesis of the composites; tensile tests, adhesion, and corrosion resistance tests were carried out according to the relevant standards; simulation and mock-up tests were carried out by ANSYS 15.0; and the materials were applied in the field to prove and validate the excellent performance of the composites through the laboratory test–simulation–field application method. It provides theoretical guidance for the application of EP as a safety marking material for offshore oil platforms.

## 2. Materials and Methods

### 2.1. Materials

Graphite powder and graphene were purchased from Henan Shengkun Chemical Products Co., Ltd., Zhengzhou, China. Concentrated sulfuric acid, potassium permanganate, anhydrous ethanol, and silane coupling agent were purchased from Tianjin Chemical Reagent Factory, Tianjin, China. EP, glass fiber, magnesia paste, aluminum hydroxide, quartz powder, and infiltrating agent were purchased from Tianyi Energy Chemical Co., Ltd., Zhengzhou, China. Among them, the purity of EP is >98%, and the particle size requires 80% distribution in the 200–500 μm interval; the glass fiber particle size is <100 mesh (about 150 μm).

### 2.2. Preparation of KGO/KGF/EP Composites

#### 2.2.1. Preparation of Graphene Oxide

Graphite powder in the mixture of concentrated sulfuric acid and KMnO_4_ was treated in three stages: low-temperature, medium-temperature, high-temperature reactions. An ultrasonic bath was used to peel the graphene oxide. The specific preparation process is as follows:

Low-temperature stage: 46 mL of concentrated sulfuric acid was added into a 500 mL beaker. Then, 1 g of graphite powder was added into the beaker in an ice bath. The solution was stirred slowly and then treated by an ultrasonic bath for 1 h, whereupon 3 g of KMnO_4_ was added into the beaker by small aliquots, each time. The solution was kept stirring and reacted for 2 h at 10 °C to obtain dark green products.

Medium-temperature stage: The above solution was treated in a water bath at 38 °C, and ultrasonic treatment was applied for 0.5 h. At this stage, KMnO_4_ begins to oxidize the graphite powder intercalated by sulfuric acid molecules, and KMnO_4_ enters the deep graphite oxide layer between the graphite layers.

High-temperature stage: 100 mL low-temperature deionized water was slowly added into the beaker, then the temperature was raised to about 95 °C. The solution was kept under moderate mechanical stirring for 0.5 h. At this stage, the oxidized graphite powder begins to hydrolyze until it is completely processed. Deionized water should be added slowly to avoid improper temperature control of the reaction liquid, which could lead to thermal decomposition of the sulfuric acid–graphite interlayer compounds. This could result in the spillage of sulfuric acid and another inserted materials from the graphite interlayer, which would seriously affect the quality of graphite oxide.

Finally, 60 mL of ice water mixture was added to stop the reaction. Then, 30% hydrogen peroxide was slowly added and stirred vigorously until the reaction liquid turned bright yellow. Afterward, an appropriate amount of hydrochloric acid solution was added. Centrifuge separation was performed until the washing liquid was neutral, after which centrifugation was stopped. The product was then subjected to ultrasonic dispersion in water, oscillation stripping, and after 50 min, centrifugation was repeated to obtain the target product.

#### 2.2.2. Preparation of KGO

A total of 0.2 g of GO was dispersed and ultrasonicated in 120 mL of anhydrous ethanol for 1 h. Afterward, 2 g of KH550 was dispersed in 80 mL anhydrous ethanol and ultrasonicated for 0.5 h. The two solutions were then mixed and stirred to form a homogeneous solution. The pH of the above solution was adjusted to 4 before heating to 80 °C in a three-neck flask for 24 h. Finally, the product was collected by centrifugation at 180 r/min for 6 h and washed with ethanol three times to remove any unreacted silane coupling agent and acid. Prepared KGO was dried at 80 °C for 6 h.

#### 2.2.3. Preparation of KGF

According to the molar ratio of KH550 to water 10:1, KH550 was configured into a 5% ethanol solution, and its pH was adjusted to about 4. Glass fiber with a mass fraction of 12% was added to the above solution, which was then heated to 50 °C under stirring for 3 h. Afterward, the product was collected by centrifugation and washed with ethanol for three times to remove the unreacted silane coupling agent and acid. KGF was then placed in the oven and dried under 80 °C for 6 h.

#### 2.2.4. Preparation of KGO/KGF/EP Materials

Graphene-based siloxane dispersions with different mass fractions were prepared through the following process: 0 g, 0.035 g, 0.105 g, 0.175 g, 0.245 g of silanized graphene oxide (based on the mass of the epoxy resin, corresponding to mass fractions of 0%, 0.1%, 0.3%, 0.5%, and 0.7% graphene-based siloxane, respectively) were weighed and ultrasonically dispersed in 35 mL of anhydrous ethanol, and ultrasonicated for 1.5 h to obtain a homogeneous and stable black dispersion. Modified glass fiber was added at wt.% to the dispersion.

The mass fraction of the KGO/KGF/EP materials are summarized in Table 1. In a typical synthesis, EP and the photoinitiator UVI16992 were mixed and stirred to form a homogeneous solution. Then, the infiltrating agent, aluminum hydroxide, quartz powder, and magnesia paste were added to the above solution at room temperature. Afterward, KGF and KGO were incorporated into the sheet-forming unit in the appropriate proportion to produce sheet to prepare KGO/KGF/EP materials.

### 2.3. Characterization

XRD patterns were measured in the range of 5–80° with a scanning rate of 4°/min using an X-ray powder diffractometer XRD-6100 (Shimadzu, Kyoto, Japan). FTIR spectra were measured within the range within 400–4000 cm^−1^ using a sensor-27 Fourier infrared spectrometer (Brooke Company, Tampa, FL, USA). Scanning electron microscopy (SEM) was conducted on a JSM-6380LV microscope (JEOL, Tokyo, Japan).

### 2.4. Corrosion Resistance Test of the Composite Materials

The corrosion resistance of the composites was evaluated according to the following criteria [15,16,17]. The tests involved exposing the materials to 3% NaCl solution at 40 °C, 5% H_2_SO_4_ solution, 5% NaOH solution, and neutral salt spray. Through these joint tests, a multi-dimensional corrosion evaluation system covering humidity and heat, salt spray, acid and alkali media is constructed to verify the applicability of KGO/KGF/EP composites in extreme marine environments. Additionally, accelerated aging tests were performed on the composites by immersing the test sheets in a 50% sulfuric acid solution at a temperature of 54 °C for 168 h to evaluate the resistance to high concentrations of sulfuric acid. The standard requires that the samples shall not blister or peel off after 7 days of immersion, no failure after 7 days of acid/alkali resistance, and no red rust after 1000 h of neutral salt spray [15,16,17].

### 2.5. Test of Mechanical Properties of Composite Materials

(1) Tensile property test (tensile strength, tensile elastic modulus, elongation at break)

According to the methodology specified in the standard [18,19], the tensile properties of the composites, including tensile stress, tensile modulus of elasticity, and tensile elongation at break, were tested using an electronic tensile testing machine. These parameters were chosen to comprehensively assess the structural reliability of the material under service loads. Tensile stress quantifies the maximum load-carrying capacity (essential for offshore safety signs subjected to extreme wind loads); modulus of elasticity reflects stiffness and resistance to deformation (safeguarding dimensional stability in dynamic marine environments); and elongation at break characterizes ductility to reduce the risk of brittle fracture. According to the standard, the modulus of elasticity is taken from the linear section of the stress–strain curve (0.05–0.25% strain), and for fiber-reinforced composites, marine-grade materials are required to have a tensile strength of ≥70 MPa and an elongation at break of ≥1.5%.

(2) Adhesion

The adhesion of the composites is evaluated according to the methods specified in the standard [20]. The instrument adopts the pulling type adhesion tester to quantify the interfacial bond strength of KGO/KGF/EP composites with metal substrates, ensuring their long-term durability in corrosive marine environments. The standard specifies a minimum threshold of 8 MPa for marine coatings. To test the performance of the composites under real-world conditions outside of the controlled environment, adhesion of the specimens was tested by mechanical pull-out tests after 90 days of immersion in dilute sulfuric acid solution (H_2_SO_4_ + NaCl 10:1) at an experimental temperature of 54 °C. At the same time, under these conditions, the composites were studied in comparison with other high-performance materials. Specimens were selected to be partially coated, and immersion experiments were carried out on the supplied specimens using 1392 coating specimens supplied by Belzona, Harrogate, UK.

(3) Water absorption

The water absorption of the composite material is tested according to the standard [21]. Water absorption is used to assess susceptibility to moisture uptake, which directly affects a material’s durability in wet environments, especially in marine applications, where high absorption can lead to swelling, distortion, or accelerated corrosion. ASTM D570-98 [21] provides standardized test methods to ensure comparable and accurate results. A threshold of 5% water absorption is specified for marine-grade materials.

### 2.6. Numerical Simulation of Wind Load Resistance of Composite Materials

Pro/E 5.0 and ANSYS 15.0 software were used for mechanical simulation analysis of safety labels of corresponding sizes. In the mechanical simulation, the thickness parameter of the marking plate is set to 2.5 mm; the spacing of the guardrail is 300 mm; and the 3 s instantaneous wind speed of 76.1 m/s is used as the wind load of the external fluid. A Boolean operation is carried out with the rigid, non-deformed bracket and the wind-fluid domain to obtain the actual fluid domain, and a symmetric unit is used for the simulation.

### 2.7. Statistical Analysis

In this study, all the experiments were repeated three times to ensure the accuracy and reproducibility of the results. The experimental data were statistically analyzed by Origin 2024, and the results were expressed as the average ± standard deviation [22].

## 3. Results and Discussion

### 3.1. FTIR Analysis

**Figure 1:**
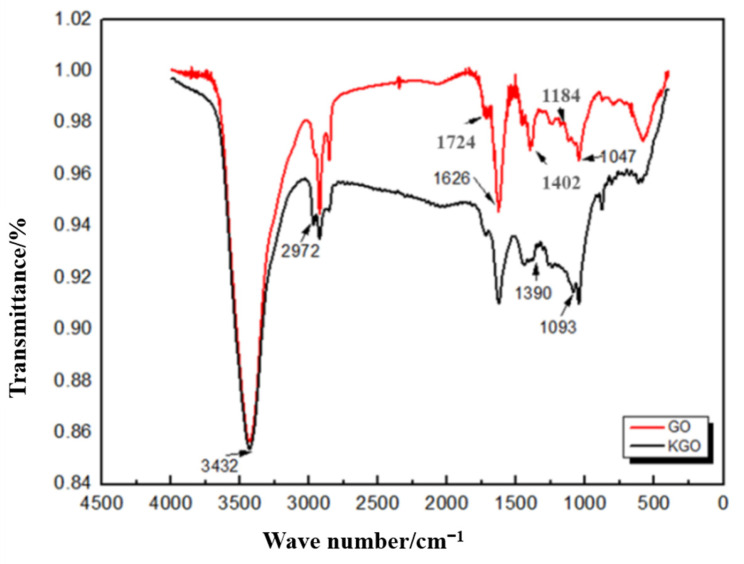
FTIR spectra of graphene oxide (GO) and graphene siloxane (KGO).

To confirm the successful modification of GO with the silane coupling agent, Fourier Transform Infrared Spectroscopy (FTIR) characterization was performed. As shown in Figure 1, the FTIR spectra of GO changed after the modification with 3-aminopropyltriethoxysilane. A new peak at 1093 cm^−1^ appeared in the KGO curve, corresponding to the Si-O-C absorption peak. The peak at 2972 cm^−1^ is ascribed to the stretching vibration absorption peak of methyl. Furthermore, the appearance of Si-H vibrational bands around 750 cm^−1^ confirms the chemical functionalization of GO by silane coupling agents. Therefore, it can be shown that the silane coupling agent was successfully modified on GO.

### 3.2. XRD Analysis

**Figure 2:**
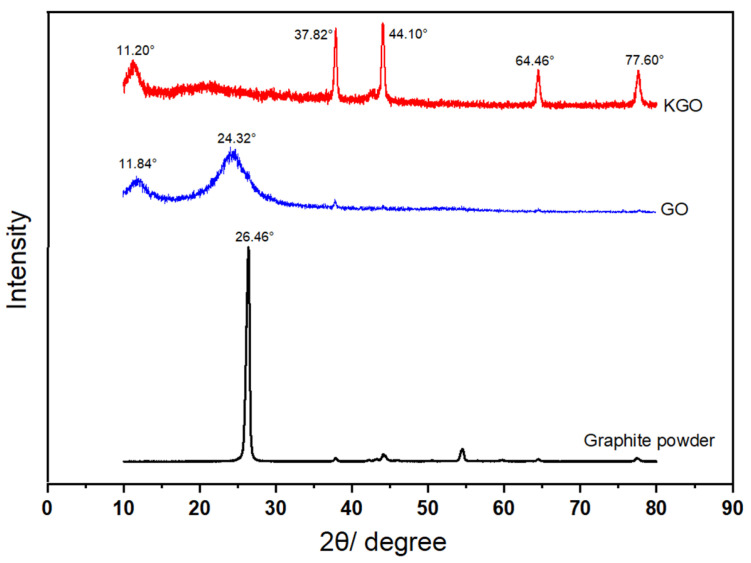
X-ray diffraction patterns of graphite powder, GO, and KGO.

In order to characterize the structure of the composites, X-ray Diffraction (XRD) characterization was carried out. Figure 2 is the XRD pattern of graphite powder, GO, and KGO, respectively. It can be seen that the diffraction peak of graphite powder is sharp, showing a typical crystalline substance. The peak at 26.46° is the characteristic peak of graphite (001), and the peak at 11.84° is the characteristic peak of GO (001). The characteristic peak of 2θ = 26.46° shifts to 24.32°, indicating an increase in graphite layer spacing and a structural change in the graphite powder following the introduction of oxygen groups, oxygen atoms, and carbon atoms. The peak at 11.20° is the characteristic peak of KGO (001), which shifts to a lower angle compared with the characteristic peak of GO (11.84°). These results show that the modification of GO by silane coupling agent is successful, which is consistent with the result of infrared spectra (Figure 1).

### 3.3. Thermogravimetric Analysis (TGA)

**Figure 3:**
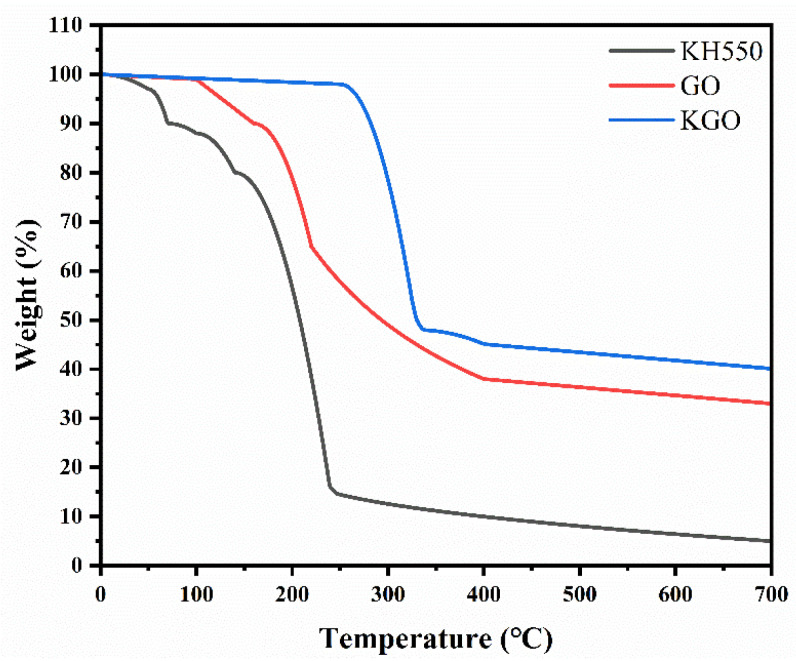
Thermogravimetric analysis graphs of KGO, GO, KH550.

TGA data of KH550-silanated graphene oxide (KGO) and unmodified GO were systematically analyzed to elucidate the enhancement of thermal stability of KGO, which is one of the key factors for its application in epoxy composites in harsh offshore environments. The results shown in Figure 3 indicate a more significant reduction in mass loss for KGO (59.9% at 700 °C) compared to GO (67%), with the delayed decomposition onset temperature increasing from 102 °C (GO) to 255 °C (KGO). The 7.1% reduction in mass loss and the significant increase in decomposition onset temperature to 153 °C is attributed to the silane cross-links formed by the KH550 grafts, which effectively delayed the thermal decomposition process, confirming the facilitating effect of silane modification. The results also indicate that the modification of GO by silane coupling agent is successful, which is consistent with the previous results.

### 3.4. Morphology Analysis of Composites

**Figure 4:**
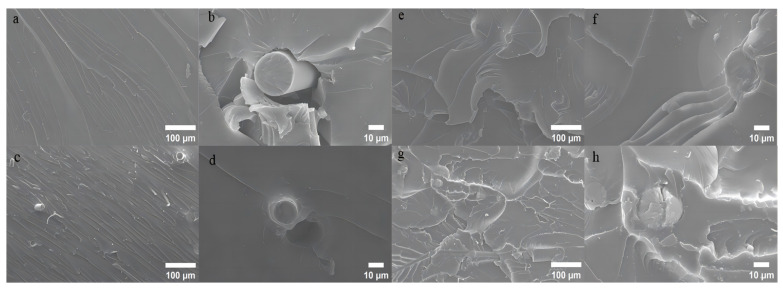
SEM images of KGO/KGF/EP composites with different contents. (**a**,**b**) KGO content is 0%; GF content is 12%. (**c**,**d**) KGO content is 0.1%; KGF content is 12%. (**e**,**f**) KGO content is 0.5%; KGF content is 12%. (**g**,**h**) KGO content is 0.7%; KGF content is 12%; EP is 55%.

The structure of the composite materials was characterized using Scanning electron microscopy (SEM), and the SEM images of the composite materials are shown in Figure 4. The KGO mass fractions were as follows: 0%, 0.1%, 0.5%, 0.7%; and both the GF and KGF mass fractions were fixed at 12%. Figure 4a,b show the SEM images of the GF/EP composite material without any modification. It can be seen from the figure that the cross-section of the material is a very smooth and brittle fracture surface with almost no visible cracks, folds, and protrusions. Figure 4c,e,g are the SEM images of the tensile section of KGO/KGF/EP composites with KGO mass fractions of 0.1%, 0.5%, and 0.7%, respectively, along with a KGF mass fraction of 12%. It can be seen that as the KGO content increases gradually, the section of the composite becomes rougher, and the folds on the section gradually increase. Brittle fracture gradually turns into a ductile fracture, because when the EP composite is subjected to external force, the addition of KGO is equivalent to adding a cushion inside the material. When the material is subjected to external force, the stress will be transferred to KGO, which reduces the stress concentration and absorbs more energy, making the material change from brittle fracture to ductile fracture [23]. It can be seen from Figure 4c,e that KGO has good dispersity in the resin matrix, and only a small amount of agglomeration occurs. However, when 0.7% of KGO was added, the viscosity of the EP matrix increased, and it can be observed that more KGO agglomeration occurs, with micro-pores appearing around it. These micro-pores are easy to be destroyed by external forces, resulting in the deterioration of the mechanical properties of the composite materials. These defects may be able to be detected quickly and easily using the eddy current model developed by Versaci et al. [24].

It can be seen that the surface of the glass fiber is smooth, and the compatibility with the EP matrix is poor, resulting in large cracks and gaps around the glass fiber when it breaks. However, for the KGO/KGF/EP composite treated with a silane coupling agent (KH550), as shown in Figure 4d, the bond between the glass fiber and the EP matrix is strengthened, and there is almost no gap between the glass fiber and the resin matrix because part of the silane coupling agent has chemical interaction with the glass fiber. It increases the compatibility and binding force between the glass fiber and the EP matrix, improving the interfacial adhesion between them and leading to excellent mechanical properties when subjected to external forces.

### 3.5. Corrosion Resistance and Mechanical Properties of Composite Materials

#### 3.5.1. Effect of KGO on Water Absorption of Composites

The water absorption rate is an important index for determining the resistance and corrosion resistance of composite materials. The lower the water absorption rate, the better the resistance and corrosion resistance. To study the influence of different KGO contents on the water absorption rate of composite materials, the test was carried out according to standard [21]. KGO/GF/EP standard samples with the addition of 0%, 0.1%, 0.3%, 0.5%, and 0.7% of KGO were prepared and recorded as sample 1, sample 2, sample 3, sample 4, and sample 5. They were weighed every 24 h. The water absorption rate of composite samples is shown in Table 2.

As can be seen from Table 2, compared with the GF/EP resin material, water absorption decreased significantly after the addition of KGO. This is because KGO acts as an effective water absorption barrier, which reduces water absorption of KGO/GF/EP. The nanoscale KGO limits the intermolecular motion around EP resin, thus slowing the relaxation of the polymer chain. As a result, the absorption and diffusion of water molecules by the resin matrix can be reduced [25]. When the amount of KGO added was 0.7%, the water absorption rate increased instead of decreasing. This is because adding too much KGO creates more voids around the resin matrix and glass fibers, and then the KGO agglomeration makes holes in the material’s structure, which leads to a shorter duration of the second stage and a higher water absorption rate.

#### 3.5.2. Effect of KGO on Corrosion Resistance of Composite Materials

**Figure 5:**
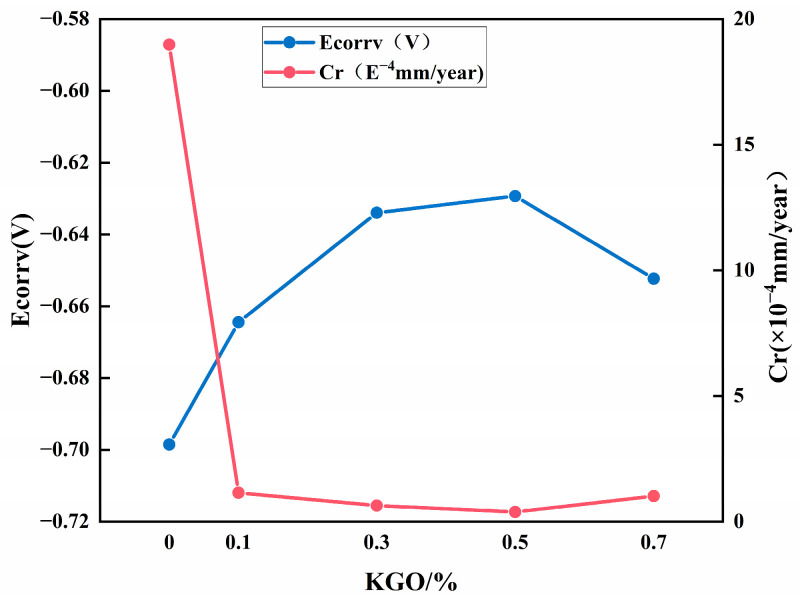
Effect of KGO on corrosion resistance of composite materials.

Corrosion potential represents the possibility of coating samples to undergo a corrosion reaction. High corrosion potential represents low corrosion possibility, slow corrosion rate, and better corrosion resistance [7]. Figure 5 shows the influence of different contents of KGO on the corrosion potential and corrosion rate of composite materials. It can be seen from the figure that with the increase in KGO content, the corrosion potential of composite materials increases first and then decreases, and the corrosion rate decreases first and then increases. When the content of KGO is 0.5%, the corrosion potential reaches −0.630 V, and the corrosion rate is 2.2 × 10^−5^ mm/year. Compared with the unmodified EP composites, the corrosion potential increased by 9.9%, and the corrosion rate decreased by 98.8%. The appropriate addition of KGO can improve the self-corrosion potential and reduce the corrosion rate of the coating material, with the best corrosion resistance observed when the addition of KGO is 0.5%.

#### 3.5.3. Effect of KGO on Adhesion Property of Composite Materials

**Figure 6:**
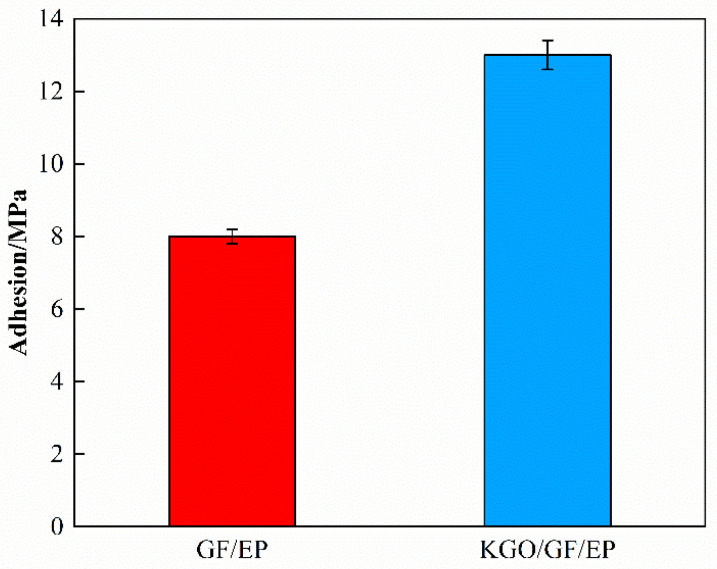
Comparison of adhesion between GF/EP and after addition of KGO.

The adhesion between the composite material and the steel substrate is another important parameter to evaluate the anti-corrosion property [26]. As shown in Figure 6, the adhesion force of GF/EP and KGO/GF/EP with steel matrix are 8.0 and 13.0 MPa, respectively. It can be seen that the addition of KGO improves the anti-corrosion performance of GF/EP composites. Due to the fact that the silane functional groups can form chemical bonds with the organic coating and metal matrix, the addition of KGO can improve the adhesion of the metal/coating interface [27,28].

#### 3.5.4. Effect of KGO on Mechanical Properties of Composites

**Figure 7:**
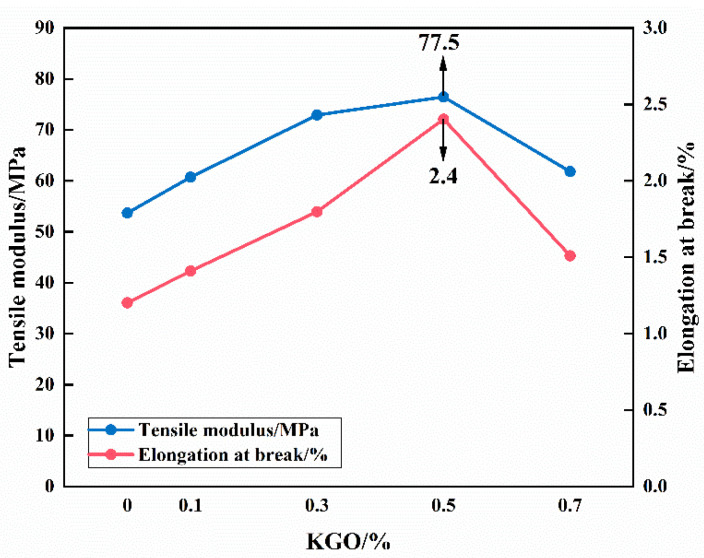
Effect of KGO content on tensile strength and elongation at break of composites.

Tensile testing of materials provides information about the strength and ductility of the material, demonstrating how much force the material withstands before failure. The two key measurements determined by tensile testing are elongation at break and tensile strength. As shown in Figure 7, the two broken lines are the measured results of tensile strength and elongation at break for materials with a fixed 12% GF content and various amounts of KGO (0%, 0.1%, 0.3%, 0.5%, and 0.7%). It can be seen from the figure that without the addition of KGO, the tensile strength and elongation at break of GF/EP composites are the lowest. After the addition of KGO, both the tensile strength and elongation at break of GF/EP composites increase rapidly. With the increase in KGO content, the tensile strength and elongation at break of GF/EP composites increase first and then decrease. When the KGO content was 0.5%, the tensile strength and elongation at break of the material reached 77.5 MPa and 2.4%, respectively, which were 42.7% and 100% higher than that of the unmodified EP. A small amount of KGO can be evenly dispersed in the EP system, while too much causes uneven distribution and agglomeration of KGO, reducing the stress transfer between the matrix and fiber subjected to tension.

**Figure 8:**
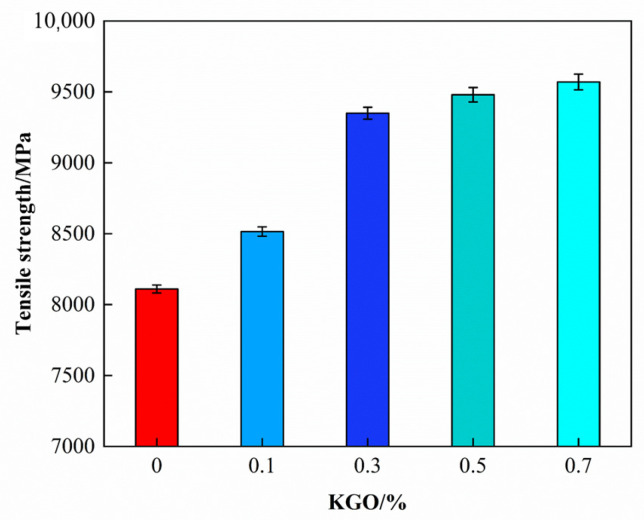
Effect of KGO content on tensile modulus of composites.

Tensile modulus is also an important factor in evaluating the performance of composites, and it increases with the addition of KGO. As shown in Figure 8, when the addition of KGO reaches 0.7%, the tensile modulus of the composite reaches its maximum value of 9570 MPa, which is 18.0% higher than that of the unmodified EP. GO has the characteristics of high strength and high modulus, and KGO also exhibits the same properties. A small amount of KGO can play a significant role in enhancing the tensile modulus.

### 3.6. Study on Mechanical Properties of Composite Materials by KGF

#### 3.6.1. Tensile Strength Test Results

**Figure 9:**
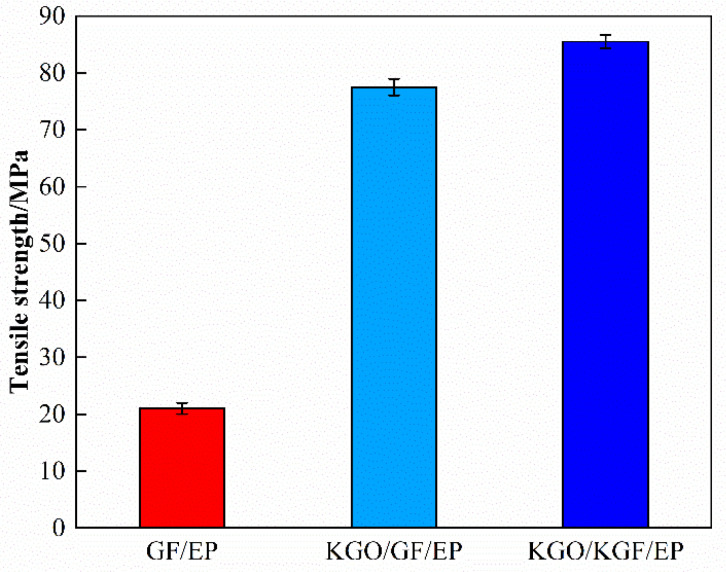
Comparison of the effects of GF and KGF on the tensile strength of composites.

The tensile strength of the composites can be further improved by adding the KGF coupling agent. As shown in the results of tensile test in Figure 9, is the tensile strength is 10.3% higher than that of KGO/GF/EP composites and 307.2% higher than that of GF/EP composites. This improvement is due to the reaction of part of the silane coupling agent with the glass fiber chemicals, increasing the compatibility and bonding between the glass fibers and EP, thereby improving the interaction between the two materials.

#### 3.6.2. Adhesion Test Results

**Figure 10:**
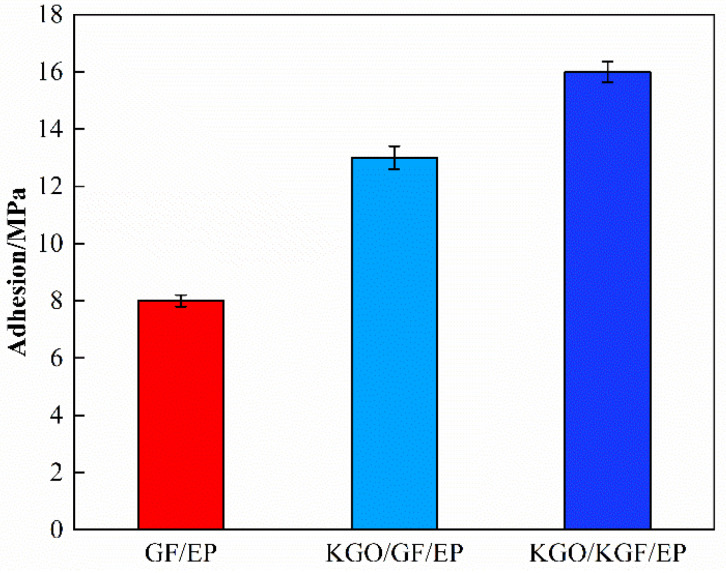
Comparison of the effects of GF and KGF on the adhesion of composites.

**Figure 11:**
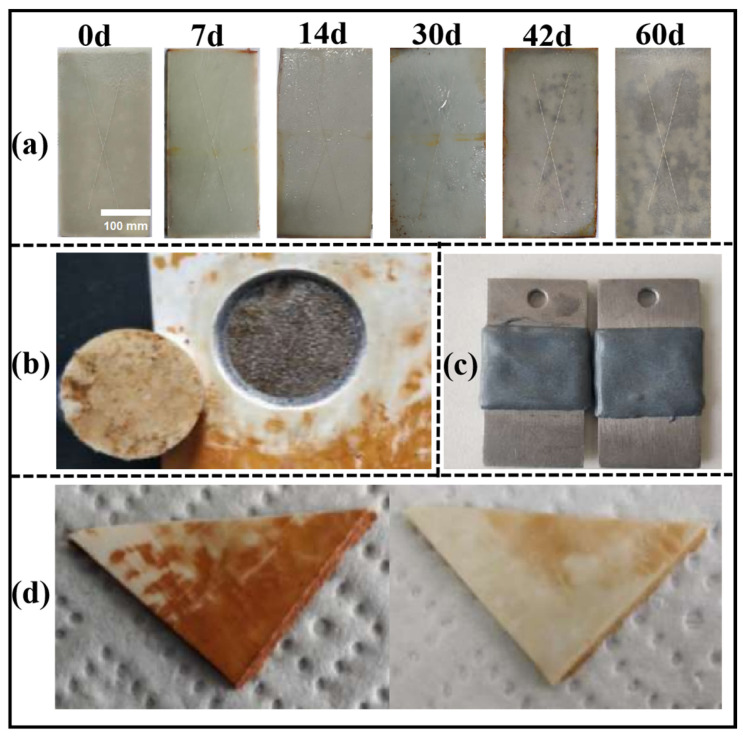
Corrosion resistance test of composite materials: (**a**) resistance to neutral salt spray for 60 days; (**b**) immersion test in 50 wt.% sulfuric acid solution; (**c**) mechanical pull-off test; (**d**) comparative immersion experiment before and after exposure.

From the previous section, it is known that the adhesion between the composite material and the steel substrate is an important parameter for evaluating the anticorrosion performance, and the silane coupling agent-modified glass fibers can further improve the adhesion of the composite material. As shown in Figure 10, the KGO/KGF/EP composites improved by 23.1% compared with KGO/GF/EP composites and by 100% compared with GF/EP composites. The adhesion of the specimens was tested at an experimental temperature of 54 °C with a solution of pH = 1 dilute sulfuric acid solution (H_2_SO_4_ + NaCl 10:1) after a soaking cycle of 90 days, as shown in Figure 11b. The bonding force was further tested using a pull-off tester, and the result was 5.35 MPa. As shown in Figure 11c, when comparing the 1392 paint specimens provided by Belzona before and after immersion, no peeling, breaking, or dissolving phenomena were found, and only a slightly golden yellow surface color appeared. Using the pull-out tester, the bonding force of these specimens was tested to be 1.9 MPa.

### 3.7. Test Results of Anticorrosive Properties of KGO/KGF/EP Composites

**Figure 12:**
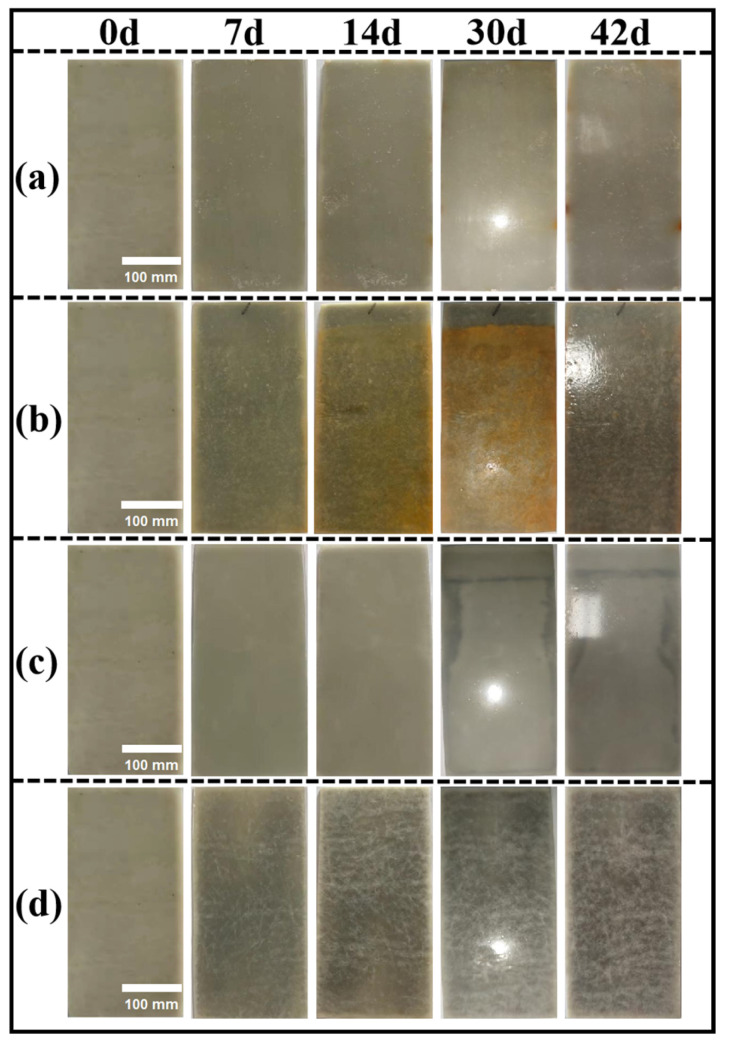
Corrosion resistance test of composite materials: (**a**) water resistance after 42 days of immersion; (**b**) resistance to heat-resistant saline solution after 42 days; (**c**) resistance to 5% sulfuric acid solution after 42 days; (**d**) resistance to 5% sodium hydroxide solution after 42 days.

According to the standards GB/T 1733 (“Determination of water resistance of paint film”) [15], GB/T 9274 (“Determination of paint and varnish resistance to liquid medium”) [16], and GB/T 1771 (“Determination of paint and varnish resistance to neutral salt spray”) [17], the following tests were conducted: water resistance, resistance to 3% NaCl solution at 40 °C, resistance to 5% H_2_SO_4_ solution, resistance to 5% NaOH solution, and resistance to neutral salt spray. The results are displayed in Figure 11 and Figure 12.

The standard requires water, acid and alkali resistance for at least 7 days [29]. However, the research results indicate that the composite material performed six times better than the standard requirements. As can be seen from Figure 12a, the composites were immersed in water for 42 d, and the surface of the samples remained smooth, without bulging, delamination, or cracking. Figure 12b–d show that when the composites were immersed in hot brine, 5% sulfuric acid solution, and 5% sodium hydroxide for 42 days, the samples exhibited similar behavior to those immersed in water. Additionally, Figure 11a demonstrates that the composite material, when immersed in hot brine for 1440 h (60 d), met the SH/T 3022-2019 [29] standard’s 1000 h corrosion resistance requirement. Only a small amount of rust appeared floating on the surface of the sample, with no signs of blistering, delamination, cracking, and other phenomena. This indicates that the composite material exhibits excellent performance, even exceeding the standard’s specifications.

As shown in Figure 11d, it can be seen that there is no obvious swelling or embrittlement when the test sheet is put into 50% sulfuric acid solution and immersed for 168 h at a temperature of 54 °C. The test sheet is also immersed in 50% sulfuric acid solution for 168 h.

### 3.8. Numerical Simulation of Wind Load

#### 3.8.1. The Establishment of Numerical Simulation

**Figure 13:**
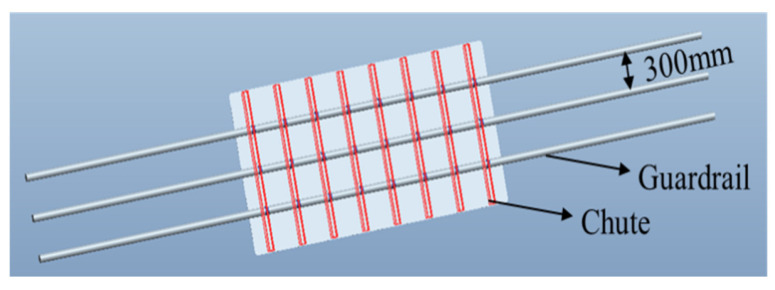
Pro/E modeling of the composite sign board with a logo size of 1200 × 2400 mm.

The installation mode is selected according to the actual application conditions. The base plate material for safety signs is usually installed on the guard rail by chute bolts on an offshore platform. Therefore, during the Pro/E modeling, the installation mode should be set according to the normal barrier spacing of 300 mm. Figure 13 shows the Pro/E three-dimensional modeling of the composite sign board. A longitudinal sliding rod is arranged, and the sliding rod and the plate are fastened by a rubber layer and screws. The influence on stress distribution, strain, and maximum deformation of the composite materials near the slide rod is studied.

The fluid–solid coupling flow in the fluid–solid coupling simulation software was established by using the fluid–solid coupling simulation model in the ANSYS Workbench platform.

#### 3.8.2. Fluid Dynamics Simulation Results and Data Analysis

**Figure 14:**
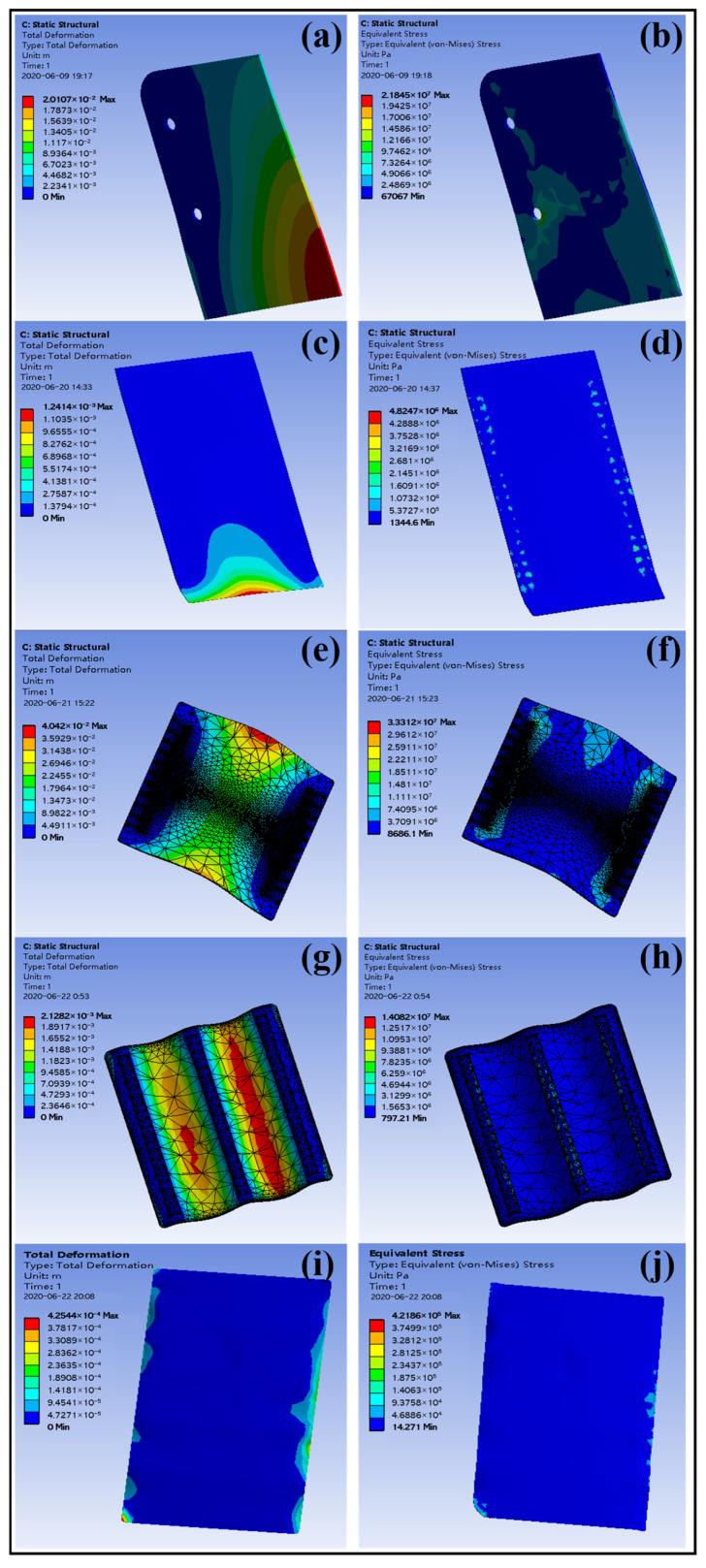
Graph of simulation results of different models: (**a**) total deformation (500 × 350 mm); (**b**) equivalent stress (500 × 350 mm); (**c**) total deformation (600 × 450 mm); (**d**) equivalent stress (600 × 450 mm); (**e**) total deformation (900 × 600 mm); (**f**) equivalent stress (900 × 600 mm); (**g**) total deformation (1100 × 900 mm); (**h**) equivalent stress (1100 × 900 mm); (**i**) total deformation (2400 × 1200 mm); (**j**) equivalent stress (2400 × 1200 mm).

The simulation analysis results for safety signs of different sizes are shown in Table 3. The fluid–structure coupling simulation results for sign plates of various specifications, subjected to a wind speed of 76.1 m/s, are shown in Figure 14. As can be seen from the simulation results, under the condition of extreme wind speed of 76.1 m/s, the fluid–structure coupling simulation results for all five sign plate specifications (without opening holes) show that the maximum stress remains within the allowable stress range. The deformation is small, and the sign plates can withstand extreme wind loads.

### 3.9. Practical Application of Composite Materials

**Figure 15:**
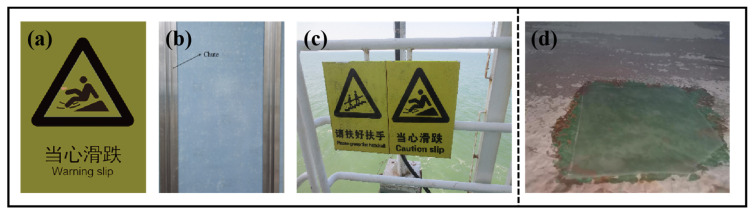
Template drawing of sign board: (**a**) front view; (**b**) back view; (**c**) 180 days later; (**d**) repair on tower wall.

We assembled the composite material into a safety mark template and installed it on an offshore oil platform to test its practical application performance. As shown in Figure 15c, after 180 days of field application, the surface of the sample remained smooth with no bubbling, delamination, cracking, rust, or other visible defects. This observation further demonstrates that the KGO/KGF/EP composite is an ideal material for offshore oil platform safety signs. The composites were also applied in other areas. For example, Figure 15d shows the application of the composites for tower wall repair. After 12 months of testing on a glass scale substrate, the test samples were retrieved and analyzed. Visually, only a change was found, with no detachment, bulging, or dissolution. Results from the EDM test showed a minimum breakdown point of 10.25 KV; and substrate adhesion was at a minimum of 3.8 MPa of the sample.

## 4. Conclusions

In this work, a breakthrough in the field of epoxy composites was achieved by integrating KH550-silanated graphene oxide (KGO) and glass fibers (KGF) to form a physical barrier that provides a synergy between mechanical robustness and corrosion resistance in maritime safety marking applications. The optimized composites (0.5 wt.% KGO, 12 wt.% KGF) have a tensile strength of 85.5 MPa and an adhesion strength of 16.0 MPa, which are 42.7% and 100% higher than conventional KGF/EP composites, respectively, while the corrosion potential (−0.630 V) has been increased by 9.9% and the corrosion rate (2.2 × 10^−5^ mm/year) by 98.8%. Numerical simulations of wind loads further validated the operational resilience of the composites under extreme wind loads (76.1 m/s), with the maximum stress (48.2 MPa) well below their tensile capacity, while 180 days of field testing confirmed their long-term durability in harsh marine environments. This study provides a simple, economical, and environmentally friendly method for the preparation of epoxy composites for offshore platforms. And the product can be subsequently used for the anticorrosion reinforcement repair of process pipelines on offshore platforms, the anticorrosion of platform decks, and the anticorrosion of thermal insulation pipe shells, which has a good prospect for promotion.

## Figures and Tables

**Table 1:** Composite formulation.

Raw Material	Mass Fraction (wt.%)
Epoxy resin	45~70
Photoinitiator UVI16992	0.08~0.35
Infiltrating agent	0.1~0.5
Magnesia paste	0.2~0.5
Aluminum hydroxide	10~20
Quartz powder	10~20
Modified glass fiber	12
Modified graphene oxide	0.5

**Table 2:** Dynamic water absorption curve of composites.

Sample	Wt. (KGO)/%	Weight of Sample Before Immersion/mg	Weight of Sample After Immersion/mg	Water Absorption Rate/%
1	0	250.5 ± 0.12	263.4 ± 0.25	5.15 ± 0.20
2	0.1%	233.4 ± 0.15	245.4 ± 0.30	5.14 ± 0.22
3	0.3%	258.9 ± 0.10	270.2 ± 0.18	4.36 ± 0.15
4	0.5%	369.7 ± 0.20	383.2 ± 0.35	3.65 ± 0.12
5	0.7%	272.3 ± 0.18	284.5 ± 0.28	4.48 ± 0.18

**Table 3:** Simulation analysis results of safety signs with different sizes.

Signboard Specification (mm × mm)	Fixed Mode (Chute Width 60 mm)	Deformation (mm)	Stress (MPa)	Allowable Stress (MPa)
500 × 350	Two side chutes fixed	20.1	21.8	Longitudinal tensile strength × 0.6 (Safety factor) = 85.6 × 0.6 = 51.4
600 × 450	Two side chutes fixed	1.24	48.2	
900 × 600	Two side chutes fixed	40.4	33.3	
1100 × 900	The three chutes are evenly distributed	2.13	14.08	
2400 × 1200	The three chutes are evenly distributed	0.425	42.2	

## Data Availability

The original contributions presented in this study are included in the article. Further inquiries can be directed to the corresponding author.
